# A Real-World Study of Patient and Physician Satisfaction With Repeated AbobotulinumtoxinA Treatment of Glabellar Lines in Chinese Patients

**DOI:** 10.1093/asjof/ojag066

**Published:** 2026-04-18

**Authors:** Jianmei Huang, Chungyung Keung, Li Ma, Jingjing Zhao, Zhiqiang Xue, Shuxian Zhang, Yuan Chen, Lin Tao, Qingyang Liu, Cecilia Persson, Inna Prygova, Weimin Song

## Abstract

**Background:**

AbobotulinumtoxinA (aboBoNT-A; Dysport^®^) is approved in China for treating glabellar lines (GLs) with fast onset of action (median: 2 days) and effectiveness up to 6 months (Chinese pivotal trial data), but real-world data remain limited.

**Objectives:**

Primary objective: to assess patient satisfaction following 3 cycles of aboBoNT-A treatment for moderate-to-severe GLs in a real-world setting. Secondary variables included effectiveness, patient and physician satisfaction in each cycle, and injection practice details.

**Methods:**

In this non-interventional, multicenter study conducted in China, participants received 3 injection cycles of aboBoNT-A in the GL area, 3 to 6 months apart, with follow-up at Week 3, before the next treatment, and at end-of-study. Assessments included satisfaction questionnaires, GL wrinkle severity, and safety.

**Results:**

At 3 weeks after the third injection, 100% of patients (*N* = 204) were satisfied with treatment (very satisfied: 71.1%; satisfied: 28.9%, primary endpoint), and physicians were very satisfied/satisfied with overall treatment outcome in 99.5% of patients. Physicians reported ≥1-grade improvement in GL severity at maximum frown in ≥90% of patients at 3 weeks in each cycle and in ≥64% at ∼5 months post-treatment (at next treatment). For ≥99% of patients, physicians would recommend aboBoNT-A to colleagues. The treatment was well tolerated, with no new safety findings.

**Conclusions:**

All patients were satisfied with aboBoNT-A GL treatment after 3 injection cycles in this Chinese real-world study. As supported by multiple outcome measures, both patients and physicians reported high satisfaction with treatment and long-lasting improvement in wrinkle severity across single or repeat cycles. Treatment was well tolerated.

**Level of Evidence: 4 (Therapeutic):**

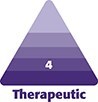

AbobotulinumtoxinA (aboBoNT-A) or Dysport*®* (Ipsen Biopharm Ltd., Wrexham, UK;/Galderma, Lausanne, Switzerland) is a botulinum toxin type A (BoNT-A) product approved in more than 80 countries worldwide for the aesthetic improvement of glabellar lines (GLs).^1,2^

In China, marketing approval for aboBoNT-A for the GL indication was granted in 2020, with a recommended dose of 50 U. In the pivotal Phase III trial conducted in China, 95% of patients were GL responders at maximum frown (none-or-mild severity, investigator-assessed) at Month 1. The median time to onset was 2 days after treatment, as noted by patients, with a third of patients reporting onset by Day 1. The none-or-mild response lasted for up to 6 months in 17% of patients treated with aboBoNT-A vs <1% for placebo (*P* = .001).^3^

In pivotal Phase III trials conducted in a Western population in the United States, aboBoNT-A 50 U demonstrated high efficacy in treating GLs, including fast onset (median: 2-3 days) and a long duration of action (4-5 months), with high patient satisfaction rates and well tolerated treatment.^4-6^

Post-marketing studies on aboBoNT-A from around the world^7-9^ support its long duration of action, through high levels of patient satisfaction for up to 6 months following a single treatment with aboBoNT-A for GLs, with results sustained across repeated treatment cycles. These studies include the APPEAL study,^7^ a longitudinal investigation conducted across 13 centers in 6 countries (Australia, Czech Republic, Kazakhstan, Lebanon, Russian Federation, and Ukraine); the DREAM study,^8^ a multicenter Phase IV trial conducted in the United States; and the ANGEL study,^9^ a prospective study carried out at 66 centers in France, Germany, Spain, and the United Kingdom.

Since aesthetic ideals may differ between different cultures and ethnicities, the present study was conducted to investigate real-world patient and physician satisfaction with aboBoNT-A in China following its approval for GL use, with a focus on long-term satisfaction after 3 repeated treatments.

## METHODS

### Study Design and Intervention

This was a prospective, longitudinal, non-interventional, multicenter study (clinicaltrials.gov NCT05089357), conducted in China across 10 sites, between November 2021 and December 2023, during the coronavirus disease 2019 (COVID-19) outbreak. The aim was to include approximately 250 patients who intended to receive long-term treatment for GLs with aboBoNT-A. Prior to study initiation, approval was received from the Independent Ethics Committee (IEC), and written informed consent was obtained from all enrolled patients, in accordance with the ethical principles of the Declaration of Helsinki. Patients paid for their own BoNT-A treatment and received only a fixed amount as reimbursement for travel costs to visit the clinic for additional (non-treatment) follow-up visits.

All patients received at least one dose of aboBoNT-A for the treatment of GLs according to clinical practice in China. As per the Chinese label, the recommended GL dose was 50 Speywood units (hereafter referred to as U) reconstituted to 0.25 mL, divided into 5 injection sites, 10 U (0.05 mL of reconstituted solution) per site, placing 2 injections into each corrugator muscle and 1 injection into the procerus muscle near the nasofrontal angle. Injections were administered intramuscularly, at right angles to the skin, by using a sterile 29 to 30 G needle.

**Figure 1.**
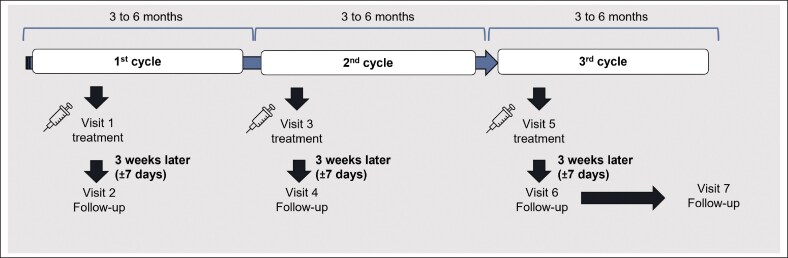
Treatment and visit schedule. The final follow-up visit (Visit 7) occurred 3 to 6 months after the third injection.

The study consisted of 3 injection cycles, each entailing an injection visit (Visits 1, 3, and 5), scheduled 3 to 6 months apart, and a follow-up visit 3 weeks after the injection (±7 days; Visits 2, 4, and 6). As generally for botulinum toxins, the China label for aboBoNT-A specifies that injections are to be at least 3 months apart. Additional follow-up assessments were performed before the injections at Visits 3 and 5, and at an end-of-study visit (Visit 7), conducted 3 to 6 months after the patient's third injection (Figure 1).

### Patients

#### Inclusion and Exclusion Criteria

Chinese adults (both males and females) up to 65 years of age, with moderate (grade 2) or severe (grade 3) GLs at maximum frown as assessed by the physician on the Investigator's validated 4-point photographic scale (grade 0—no wrinkles, grade 1—mild wrinkles, grade 2—moderate wrinkles, grade 3—severe wrinkles), were eligible for the study. In addition, patients were required to be seeking long-term treatment for their GLs before and independently of the study, and the physician must have intended to use aboBoNT-A for treatment before the patient's enrollment in the study. The main exclusion criteria included hypersensitivity to aboBoNT-A or its excipients, contraindications to aboBoNT-A as specified in the approved leaflet in China, infection in the proposed treatment area, and previous treatment failure with other toxins. Women who were pregnant, breastfeeding, or intending to become pregnant during the study were also excluded, as were individuals who had participated in a clinical trial within 30 days before the study.

### Endpoints and Assessments

The primary objective of the study was to evaluate patient satisfaction following 3 injection cycles of aboBoNT-A for the treatment of GLs. At Visit 6 (ie, 3 weeks ±7 days, after the third injection), patients were asked, “What is your overall satisfaction after 3 treatment cycles with aboBoNT-A?” The response options were *“*very satisfied,” “satisfied,” “dissatisfied,” and “very dissatisfied.” The primary endpoint evaluated the proportion of patients in each response category.

As a secondary objective, physician overall satisfaction after 3 injection cycles with aboBoNT-A was evaluated based on the proportion of responses in each category (very satisfied, satisfied, dissatisfied, very dissatisfied) at Visit 6 (3 weeks ±7 days after the third injection). Other secondary objectives included assessment of patient and physician satisfaction at each visit, and evaluation of effectiveness of aboBoNT-A in treating GLs, measured by responder rates of ≥1-grade improvement in GL wrinkle severity from baseline at Visits 2 through 7. GL wrinkle severity was assessed by the physician using the Investigator's validated 4-point photographic scale at maximum frown and at rest^3^ and by the patient using a 4-point categorical scale (no wrinkles, mild wrinkles, moderate wrinkles, severe wrinkles) at maximum frown. Lastly, injection practice details were collected at each treatment visit, including a description of the muscles injected, the dose and volume (in total and per injection point), and the mean retreatment interval between injections, as recorded at Visits 1, 3, and 5. Safety was assessed by adverse events (AEs) collected throughout the clinical study.

### Statistical Analysis

All data were analyzed descriptively, with effectiveness endpoints summarized based on the Per Protocol Set (PPS) and safety data summarized based on the Safety Set. The PPS included all enrolled patients who were treated at least once with aboBoNT-A and who had evaluable primary effectiveness endpoints, overall good compliance, and no major protocol deviations during the study. The Safety Set included all patients who received at least one dose of aboBoNT-A treatment and had available safety data.

## RESULTS

### Patient Disposition and Baseline Characteristics

The study screened and enrolled 250 patients, all of whom received aboBoNT-A and were included in the Safety Set. A total of 216 patients (86.4%) completed the study. Among the 34 patients (13.6%) who did not complete the study, the most frequently reported reason for discontinuation was withdrawal of consent (67.6%). The PPS included 204 patients, with 34 patients excluded due to missing primary endpoint and 12 patients excluded due to a major protocol deviation, ie, severely delayed visit for the primary assessment (>21 days out of visit window). The majority of patients in the PPS (92.6%) identified as Han Chinese and most (84.3%) were female. The mean age of the patients was 37.8 years (SD: 9.48 years; range: 21-63 years; Table 1).

**Table 1.** Demographics, Per Protocol Set

Age, years	Mean (SD)	37.8 (9.48)
	Min, Max	21, 63
Sex, *n* (%)	Male	32 (15.7%)
	Female	172 (84.3%)
Nation, *n* (%)	Han	189 (92.6%)
	Other	15 (7.4%)

*n*, number of subjects.

### Treatments

The most common reason for treatment, reported by 93.6% of patients, was a “personal wish to improve my appearance or attractiveness.” The number of patients treated per cycle was 250 (cycle 1), 235 (cycle 2), and 224 (cycle 3) in the Safety Set, and 204 patients in each cycle in the PPS. The mean retreatment interval in the PPS was approximately 5 months (152.6 days [SD: 62.54 days] between the first and second injections, and 156.4 days [SD: 49.63 days] between the second and third injections).

At each of the 3 injection cycles, patients in the PPS were injected with a median dose of 50 U, with 8 U as the lowest reported injected dose at any treatment cycle and 60 U as the highest injected dose and 75% of patients receiving a dose of 46 U or higher, based on the lower quartile limits at each treatment, Table 2. The median volume of reconstituted solution was 0.25 mL at each treatment cycle, with min-max ranges from 0.05 to 0.50 mL, Table 2. Most patients received treatment at 5 injection points (93.1% of patients at the first injection visit, 89.7% at the second injection visit, and 87.3% at the third injection visit). The remaining patients received injections at 2 to 4 points only (Table 2).

**Table 2.** Injection Details, PPS

	Injection 1 Visit 1	Injection 2 Visit 3	Injection 3 Visit 5
Total injected dose, U			
*n* (nmiss)	204 (0)	204 (0)	204 (0)
Mean (SD)	45.95 (7.865)	46.53 (8.064)	46.01 (9.620)
Median	50.00	50.00	50.00
Q1, Q3	46.00, 50.00	50.00, 50.00	50.00, 50.00
Min, max	8.0, 50.0	20.0, 50.0	10.2, 60.0
Total injected volume, mL			
*n* (nmiss)	204 (0)	204 (0)	204 (0)
Mean (SD)	0.2399 (0.0524)	0.2405 (0.0599)	0.2374 (0.0663)
Median	0.2500	0.2500	0.2500
Q1, Q3	0.2500, 0.2500	0.2500, 0.2500	0.2500, 0.2500
Min, max	0.080, 0.500	0.100, 0.500	0.051, 0.500
Number of injection points, *n* subjects (%)			
5	190 (93.1%)	183 (89.7%)	178 (87.3%)
4	2 (1.0%)	0	1 (0.5%)
3	12 (5.9%)	19 (9.3%)	23 (11.3%)
2	0	2 (1.0%)	2 (1.0%)

*n*, number of subjects; nmiss, number of subjects who missed the visit; Q1, first quartile; Q3, third quartile.

### Effectiveness Outcomes

#### Primary Endpoint: Overall Patient Satisfaction After 3 Injection Cycles

**Figure 2.**
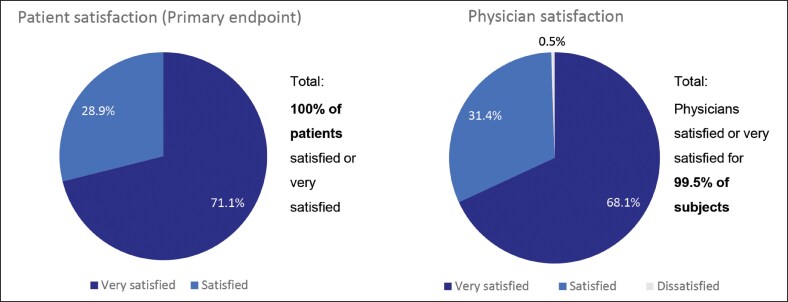
Patient and physician overall satisfaction at week 3 after the third injection cycle, *N* = 204, per protocol set. *N*, number of subjects.

At 3 weeks after the third injection, all patients (100%, *N* = 204, PPS) were “very satisfied” (71.1%) or “satisfied” (28.9%) with the overall outcome after 3 injection cycles with aboBoNT-A for the treatment of GLs (Figure 2).

### Secondary Endpoints

#### Overall Physician Satisfaction After 3 Injection Cycles

The physicians also reported high overall satisfaction at 3 weeks after the third injection and were satisfied/very satisfied with the overall outcome in 99.5% of patients (“very satisfied,” 68.1%, or “satisfied,” 31.4%) (Figure 2).

### Patient Satisfaction After Each Injection Cycle

**Figure 3.**
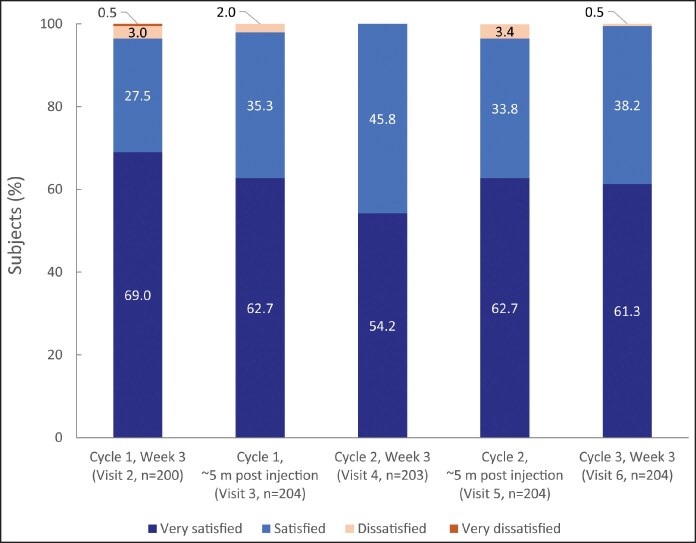
Patient satisfaction with the aesthetic outcome in the injected area after treatment, per protocol set. *n*, number of subjects at each visit.

At the Week 3 visit in all treatment cycles, nearly all patients, 96.5% (cycle 1), 100% (cycle 2), and 99.5% (cycle 3), were “very satisfied” or “satisfied” with the aesthetic outcome in the injected area. The high satisfaction with aesthetic outcome (≥97%) was also sustained until the next treatment occasion, ie, for approximately 5 months (153 or 156 days) between treatments (Figure 3) and until the end-of-study visit at 132 days after the third injection (97.5%).

Almost all patients (98.0%-100%) were “very satisfied” or “satisfied” with the comfort of the injection, when asked at Week 3 after injection in each treatment cycle.

**Figure 4.**
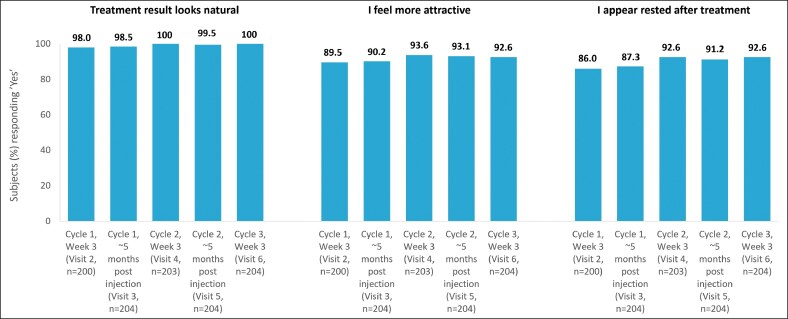
Patient satisfaction questionnaire, per protocol set; proportion of patients reporting a natural look, feeling more attractive and appearing rested after each treatment. *n*, number of subjects at each visit.

Throughout the study, patients also felt that they appeared rested (≥86%), felt more attractive after treatment (≥90%), and that the treatment outcome looked natural (≥98%) (Figure 4). For most patients (94.5%-99.0%), the treatment met or exceeded their expectations, and 98.0% to 99.5% of patients felt better about themselves after treatment. Most patients reported positive feedback about their looks from family, friends, and colleagues, with gradual increases in the positive responses observed at each visit (77.5%-88.2% across visits), and high rates of positive responses maintained until the end-of-study visit (87.0%). At the end-of-study visit (Visit 7), most patients stated that they would be happy to receive the same treatment again (98.0%) and would recommend the treatment to family or friends (97.5%).

### Patient Assessment of AboBoNT-A Compared With Prior Treatment with Another BoNT-A

Before treatment at Visit 1, 41% of patients (83/204 patients, PPS) reported previous use of botulinum toxins for the treatment of GLs. Among these patients, a consistent majority (72.7%-78.1% of patients per visit) reported “much better” or “better” aesthetic results with aboBoNT-A than with prior botulinum toxin treatments. Additionally, at the final visit, 64.8% of these patients responded that the results from aboBoNT-A treatment lasted longer than prior treatments with another product.

### Physician Satisfaction in Each Treatment Cycle

**Figure 5.**
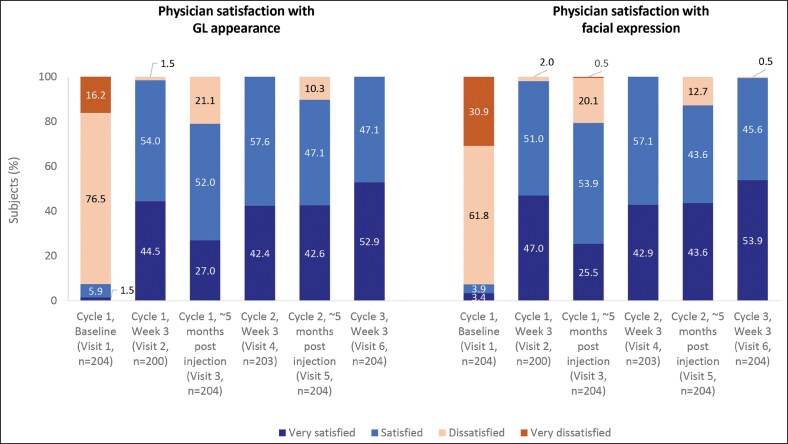
Physician satisfaction with patients' GL appearance and facial expression, per protocol set. GL, Glabellar line; *n*, number of subjects at each visit.

The proportion of patients for whom physicians were “satisfied” or “very satisfied” with GL appearance and facial expression increased from 7.4% before the first treatment to ≥98% at 3 weeks after each treatment. Furthermore, for ≥79% of patients, physicians were still satisfied/very satisfied with the GL appearance and facial expression until the next treatment (ie, for approximately 5 months; 153 days or 156 days after each injection) (Figure 5). Across all post-treatment visits, physicians reported natural-looking results in 99.0% to 100% of patients and that 95.1% to 98.0% looked more attractive, and 97.5% to 100% looked younger following treatment.

For 99.0% of patients, physicians stated at the end-of-study visit (Visit 7) that they would recommend aboBoNT-A to colleagues or other physicians.

### Improvement in Glabellar Line Severity at Maximum Frown and at Rest

The GL severity improvement responder rates reported by physicians were consistently high at maximum frown in all 3 treatment cycles. At 3 weeks post-treatment, 94.6%, 89.7%, and 96.1% of patients had ≥1-grade improvement from baseline in GL severity and the ≥1-grade improvement rates remained at 63.7% in cycles 1 and 2 until the next injection visit, for on average 5 months post-injection (153 days or 156 days between treatments).

Patient-reported results were consistent with physician assessments, with 91.7%, 85.3%, and 91.7% of patients reporting a ≥1-grade improvement from baseline in GL severity at maximum frown at 3 weeks after each treatment, and 54.9% and 59.8% of patients reporting ≥1-grade improvement until the second and third treatment visits (on average 5 months; 153 days or 156 days).

At the end-of-study visit, approximately 132 days after the third injection, a ≥ 1-grade improvement in GL severity at maximum frown was reported in 77.0% of patients by physicians and in 70.1% by the patients themselves.

Physicians also assessed GL severity of patients at rest, which showed high rates of ≥1-grade improvement from baseline at 3 weeks after each treatment (81.9%, 80.9%, and 84.3%, at Visits 2, 4, and 6), as well as at approximately 5 months (153 days or 156 days) after each injection (50.5% and 60.8% of patients, pretreatment at Visits 3 and 5) and at the end-of-study visit, approximately 132 days after the third injection (66.2% of patients).

### Safety

Five patients (2.0%) had treatment-related AEs, either judged as related to the product (dizziness in 2 patients) or the injection-procedure (dizziness, myalgia at the injection site, or injection site discomfort in 1 patient each). All events were mild or moderate, and non-serious and none led to discontinuation from the study. No eyelid or brow ptosis was reported.

## DISCUSSION

Achieving patient satisfaction is a key goal for aesthetic treatments and assessing the patient-perspective following GL treatment with BoNT-A has therefore gained increasing interest in recent years.^10^ For aboBoNT-A, prior studies with such focus have included the APPEAL study (2018),^7^ an international real-world study across different continents, which followed patients over ≥3 cycles of GL treatment; the DREAM study (2021),^8^ a US-based study investigating patient satisfaction with twice-yearly treatment over 2 treatment cycles, and the ANGEL study (2015),^9^ a European non-interventional study, which assessed patient satisfaction at 3 weeks and 4 months after a single GL treatment with aboBoNT-A. The present study was the first to assess aboBoNT-A real-world treatments in China on a larger scale. Aesthetic preferences and ideals may be influenced by ethnicity and culture,^11^ and there are also data indicating possible differences in facial muscles between populations from different geographical regions.^12^ Since these factors could impact both the outcomes of aesthetic BoNT-A treatments and the perception of and satisfaction with such treatments, outcomes in various populations, including Asians, are important to investigate.

From the patient perspective, the high satisfaction reported in the present study among Chinese patients—100% overall satisfaction reported after the third injection and 96.5% to 100% satisfaction with aesthetic outcome reported at Week 3 in each cycle, aligns with previous studies focusing on assessing patient satisfaction both after a single GL treatment cycle (APPEAL, DREAM, ANGEL)^7-9^ and after repeated treatment at 5- to 6-month intervals (APPEAL and DREAM).^7,8^ The Chinese patients in the present study (≥98%) also found that aboBoNT-A GL treatment achieved natural-looking results, similar to the results from the ANGEL, APPEAL, and DREAM studies.^7-9^

Physician assessments in the present study consistently showed high satisfaction rates with GL appearance of the Chinese patients after each injection cycle (for 98.5%-100% of patients) and natural-looking results for ≥99.0% of their patients. These results are aligned with APPEAL study results,^7^ which also measured physician satisfaction and demonstrated high rates of satisfaction with GL appearance (in 98% of patients) at Week 3 after the first injection cycle, as well as after the third injection (100%).^7^

In the present study, the effectiveness in terms of GL severity improvement rates at maximum frown was also high following aboBoNT-A treatment in Chinese patients. At 3 weeks after each injection, ≥85.3% of patients had ≥1-grade improvement from baseline in GL severity, as assessed by both patients and investigators, and ≥54.9% had ≥1-grade improvement from baseline until the next treatment (approximately 5 months). Overall, our results in the Chinese population are consistent with those reported in previous Phase II-IV studies investigating ≥1-grade improvement following treatment with aboBoNT-A.^5,10^  ^,13-15^ In the APPEAL study,^7^ which had a similar study design as the Chinese real-world study, patients had an average retreatment interval of approximately 5 months, similar to the present study; however, effectiveness was not measured in terms of ≥1-grade improvement. The DREAM study also reported similar results in a US population, showing ≥1-grade improvement rates of ≥98% at Month 1 in both treatment cycles.^8^ At 6 months after treatment, the rates were 37% as assessed by investigators and 28% by self-assessment in cycle 1% and 50% and 49%, in cycle 2.^8^ Approximately two-thirds of patients in the China study with prior experience of BoNT-A treatments responded that the results obtained with aboBoNT-A lasted longer than with other BoNT-A treatments. Treatment with aboBoNT-A in the present study was well tolerated, with no new safety findings. Five patients (2.0%) experienced treatment-related AEs and no ptosis was reported.

Limitations of this study, and potential sources of bias, include its open-label design, and the absence of a control group or blinded evaluators, and that comparisons to prior BoNT-A treatments in the patient questionnaire may be subject to recall bias. However, for the purpose of understanding patients' experiences, particularly satisfaction with repeated treatment with aboBoNT-A for GLs in a Chinese population in a real-world setting, the study provides valuable evidence to complement prior Phase III trial efficacy data.^3^

## CONCLUSIONS

In this real-world clinical study, all patients were satisfied with the overall outcome after 3 injection cycles with aboBoNT-A for GLs. The study showed high levels of patient and physician satisfaction and long-lasting improvement in GLs in Chinese patients after single- or repeated aboBoNT-A treatments, with consistent results across all 3 treatment cycles. The treatment was well tolerated, with no new safety findings and the findings in the Chinese patients were consistent with other real-world studies conducted with aboBoNT-A in different continents.
